# Incidence and Demographic Profile of Ewings Sarcoma: Experience From a Tertiary Care Hospital

**DOI:** 10.7759/cureus.18339

**Published:** 2021-09-28

**Authors:** Deeksha Muralidhar, Gramani Arumugam Vasugi, Sandhya Sundaram

**Affiliations:** 1 Pathology, Sri Ramachandra Institute of Higher Education and Research (SRIHER), Chennai, IND

**Keywords:** ewsr1-fli1, immunohistochemistry, theragnosis, chromosomal translocation, pathobiology

## Abstract

Introduction

Ewing sarcoma (ES) family of tumors (EFT) represents the second most common primary bone malignancy affecting children and adolescents after osteosarcoma. The tumor is characteristically associated with a chromosomal translocation resulting in a fusion transcript (EWSR1-FLI1). However, new molecular techniques have significantly transformed our understanding of this rare disease. The present study aims to analyze the incidence and demographic profile of Ewings sarcoma with an insight into the recent updates of the Ewing sarcoma (ES) family of tumors (EFT).

Materials and methods

All cases of Ewings sarcoma/peripheral neuroectodermal tumor (PNET) presented at a tertiary care center in South India from January 2010-December 2020 were included in this study. The demographic profile and patient details were obtained from the medical records section. Pathology reports of the included cases were retrieved, and associated factors were analyzed, including immunohistochemical studies and molecular workup.

Results

Out of the 58 cases included in the study, 30 cases (52%) were children and adolescents (< 20 years) and the rest 28 cases (48%) were adults. The mean age was 22.56. Female preponderance was noted, with 32 cases (56%) being females and 26 cases (44%) were males. The location of the tumor was variable. Twenty-five (25) cases (44%) were found in bones such as the clavicle, tibia, and mandible. Seven cases were seen on the anterior chest wall. Other sites included the oropharynx, lungs, endobronchial, infrascapular region, retroperitoneum, and thighs. One case presented as metastatic Ewings sarcoma with divergent differentiation in lungs with the primary site of the tumor being the right humerus. Immunohistochemical (IHC) studies were done on 55 of the 58 tumors. Forty-six (46) cases (80.9%) were CD99 positive and 41 cases(71.4%) were FLI-1 positive. Eleven (11) cases were both CD 99 and FLI-1 positive. NKX2.2, a recent IHC marker, was positive in six cases.

Conclusion

Ewings sarcoma has a peak incidence in the second decade of life with a propensity toward the axial skeletal location. Understanding the pathobiology and molecular updates of ES is significant to differentiate them from aggressive round cell sarcomas. They not only aid in predicting the prognosis of these aggressive tumors but also guide in therapy.

## Introduction

Ewing sarcoma (ES) and peripheral primitive neuroectodermal tumor (PNET) were originally described as distinct clinicopathologic entities. In 1918, Stout described a tumor of the ulnar nerve with gross features of sarcoma, which on histology showed small round blue cells focally arranged as rosettes; this entity was subsequently designated as neuroepithelioma and then PNET [1]. ES was described by James Ewing in 1921 as an undifferentiated tumor involving the diaphysis of long bones and in contrast to osteosarcoma, they are radiation sensitive. Although the common primary site being bone, ES was also reported to arise in soft tissue (extraosseous Ewing sarcoma (EES))[2]. However, over the last few decades, it is clear that these entities comprise the same spectrum of neoplastic lesions known as the Ewing sarcoma family of tumors (EFT). Characteristic histology with immunohistochemical expression of CD99 and FLI1 after the exclusion of other small round blue cell tumors is considered diagnostic of ES. It can develop in almost any bone or soft tissue but the most common site is in a flat or long bone, and patients typically present with localized pain and swelling.

Although rare, the Ewing sarcoma family of tumors (EFT) represent the second most common primary bone malignancy affecting children and adolescents, after osteosarcoma [3]. In 85 to 90 percent of cases of EFT, a recurrent chromosomal translocation, t(11;22)(q24;q12), fuses the 5' portion of the EWSR1 gene on chromosome 22 to the 3' portion of the FLI1 gene on chromosome 11. This can be detected using fluorescence in situ hybridization [4]. ES can exhibit a variable degree of neural differentiation; this is usually subtle and often detected only by immunohistochemical staining for markers of neural differentiation or by ultrastructural examination. Although no routinely used histochemical or immunohistochemical stain can positively distinguish EFT from other undifferentiated small round cell tumors of childhood, the vast majority of EFT express high levels of a cell surface glycoprotein that is encoded by the CD99 (MIC2X) gene [5-6].

New molecular techniques like next-generation sequencing (NGS) have significantly transformed our understanding of this rare disease. The present study aims to analyze the incidence and demographic profile of Ewings sarcoma with an insight into the recent updates of the Ewing sarcoma (ES) family of tumors (EFT).

## Materials and methods

All cases of Ewings sarcoma/PNET that presented in a tertiary care center in South India from January 2010-December 2020 were included in this study. Patients of all age groups with a diagnostically proven osseous and extraosseous Ewings sarcoma were included in the study. Cases with histological suspicion of Ewings sarcoma but not proved immunohistochemically were excluded from the study. The demographic profile and patient details were obtained from the medical records section. Pathology reports of the included cases were retrieved and analyzed for factors, including immunohistochemical studies and molecular workup. Institutional ethical clearance (CSP-MED/12/DEC/05/48) was obtained for the study.

A structured proforma was designed to record the clinical details, which included age, gender, presenting symptoms, site of the tumor, radiological findings (chest X-ray, computerized tomogram (CT), positron emission tomogram (PET) scan), and diagnostic investigations (ultrasound or computed tomography (CT)‑guided biopsy), pathological, immunohistochemical, and molecular characteristics [t(11:22) (q24:q12)] of the tumor, and other laboratory investigations and treatment modalities. The patients were categorized according to their age ( >20, 20-40, and <40 years) and based on the primary site of the tumor. Histopathological and immunohistochemical slides belonging to all the study cases were retrieved and analyzed. IHC markers of other small round blue cell tumors like CD-45, Desmin, Myogenin, SAT-B2, etc. were done to differentiate ES from them. Molecular testing by NGS was performed for a few of the cases. The grading and staging of excision specimens were based on the FNCLCC (French Federation of Cancer Centers Sarcoma Group) grading system. Variables following normal distribution were expressed as mean (standard deviation), and variables that followed skewed distribution were expressed as median.

## Results

**Figure 1:**
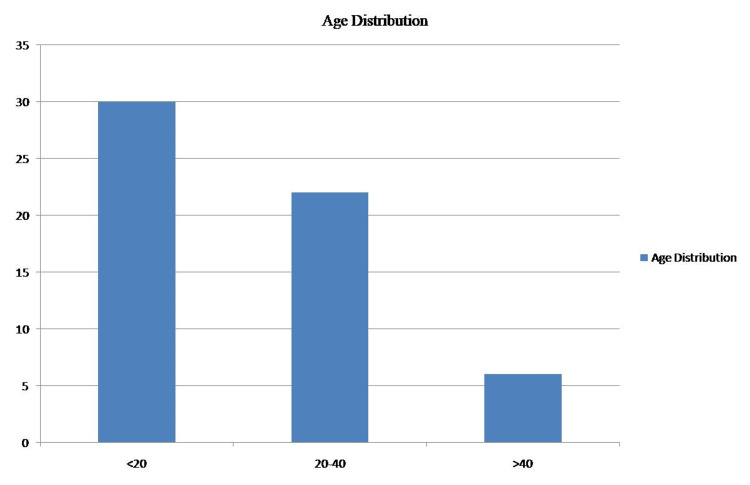
Age distribution

**Figure 2:**
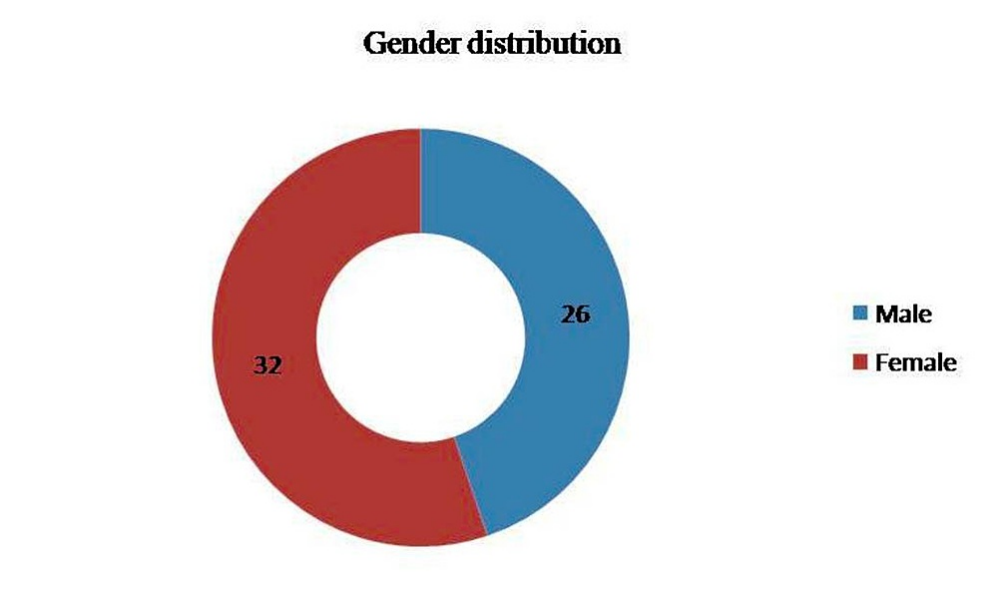
Gender distribution

**Figure 3:**
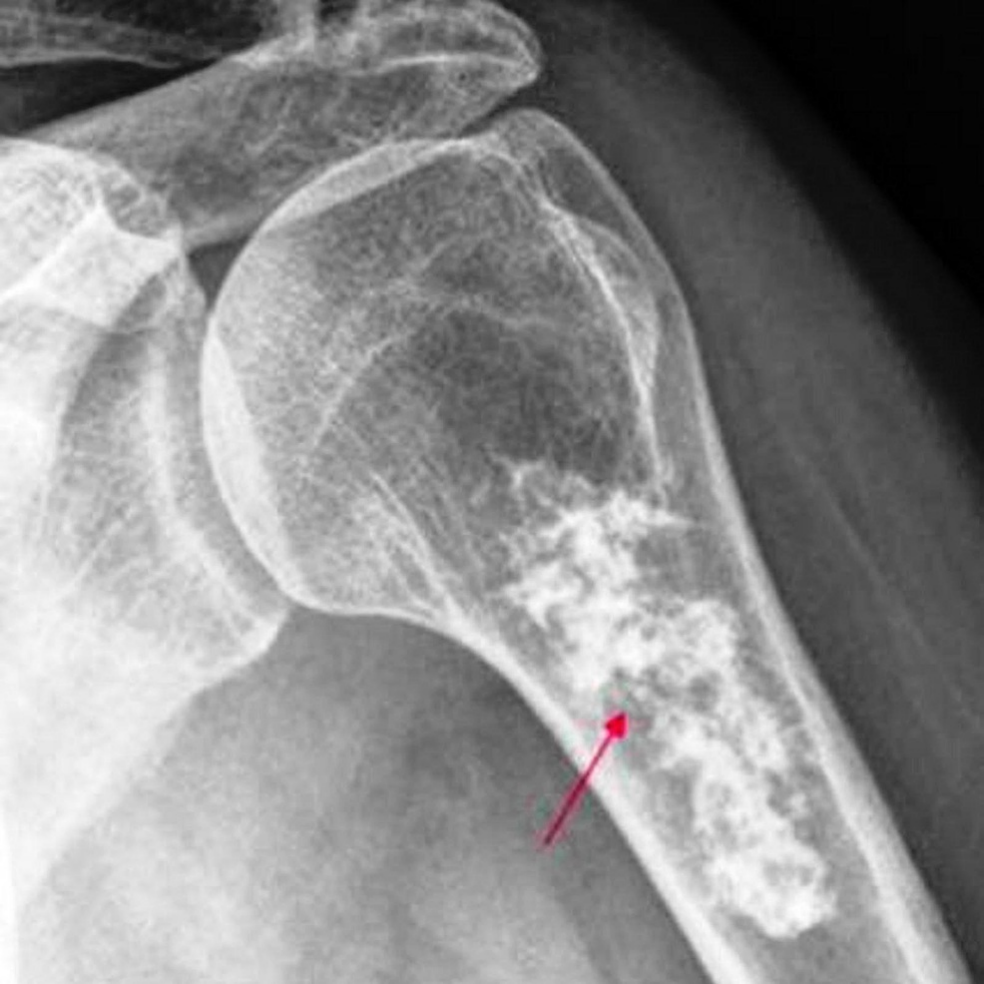
Radiology Radiology of the hypodense lesion in humerus involving the diaphysis and metaphysis

**Figure 4:**
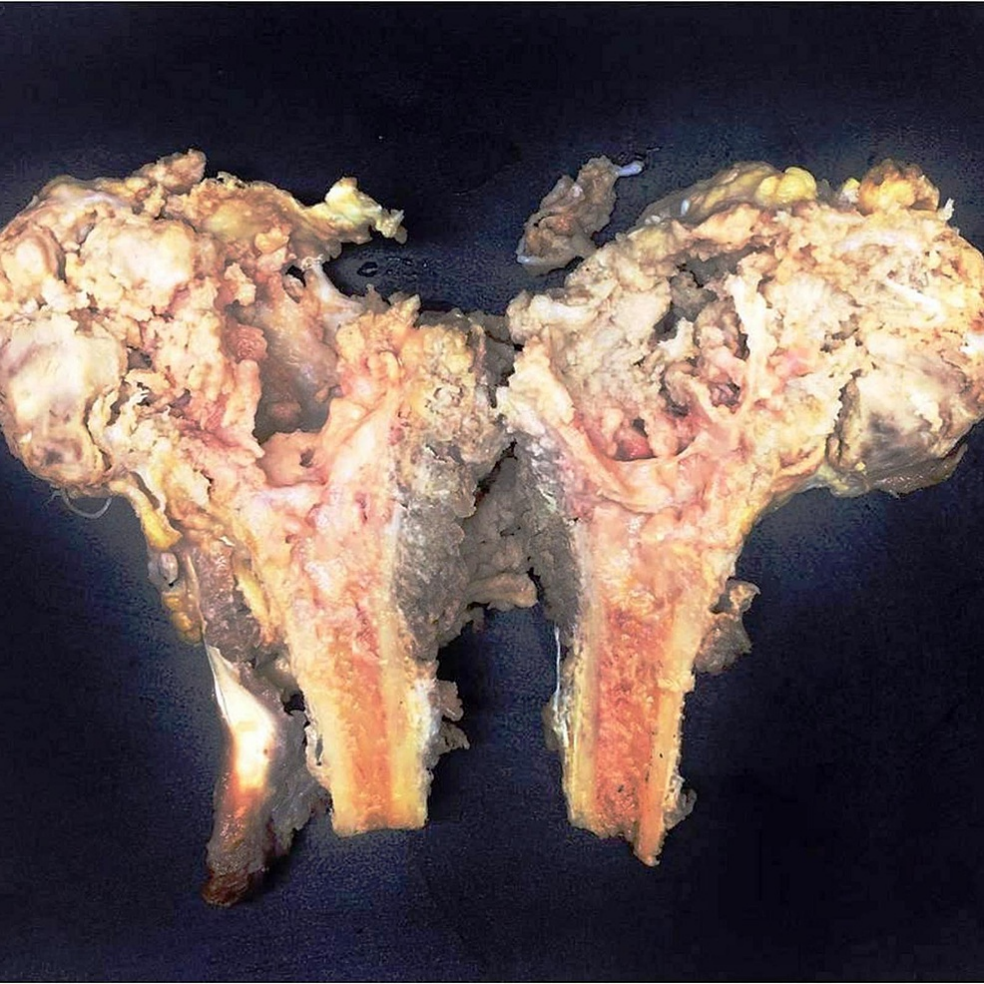
Gross Gray white friable lesion in the diaphysis extending to the metaphysis, epiphysis, and adjacent soft tissue

**Figure 5:**
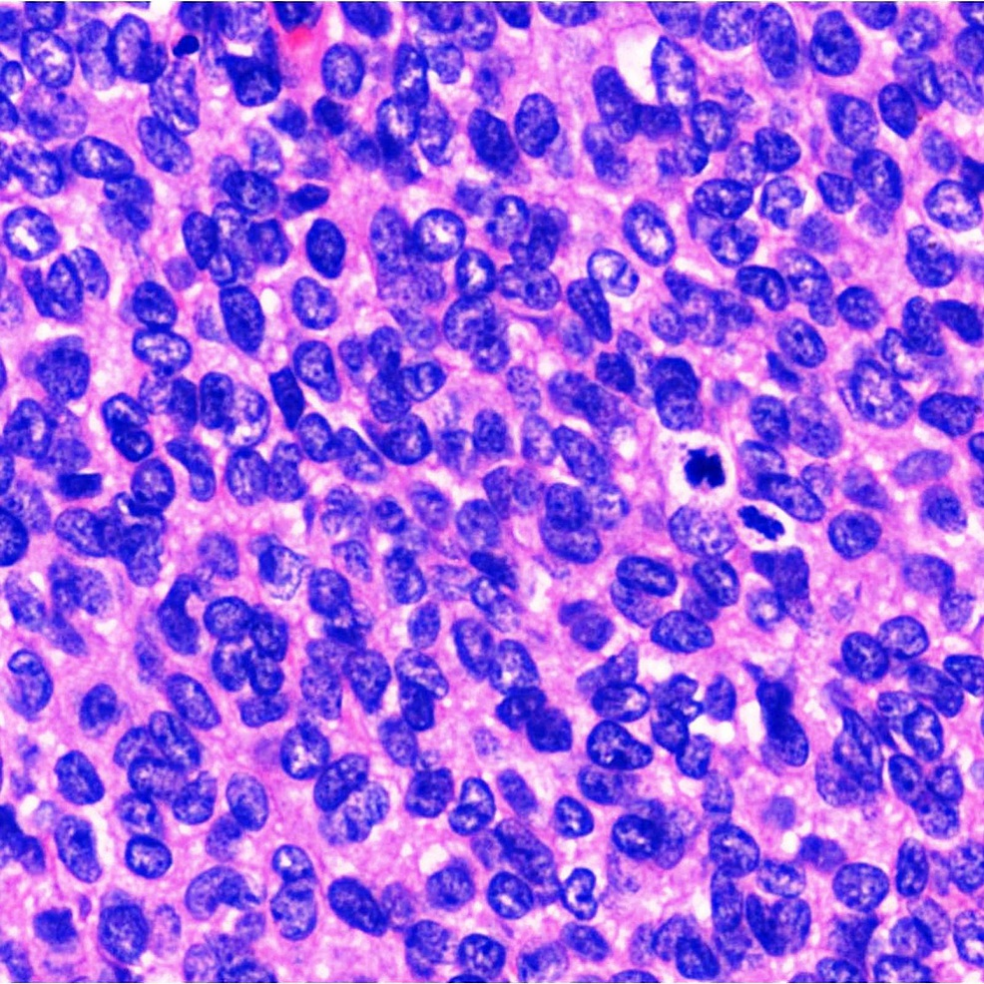
Histology Histology showing sheets of small round blue cells with an indistinct cell membrane and hyperchromatic nucleus (H&E x 200X)

**Figure 6:**
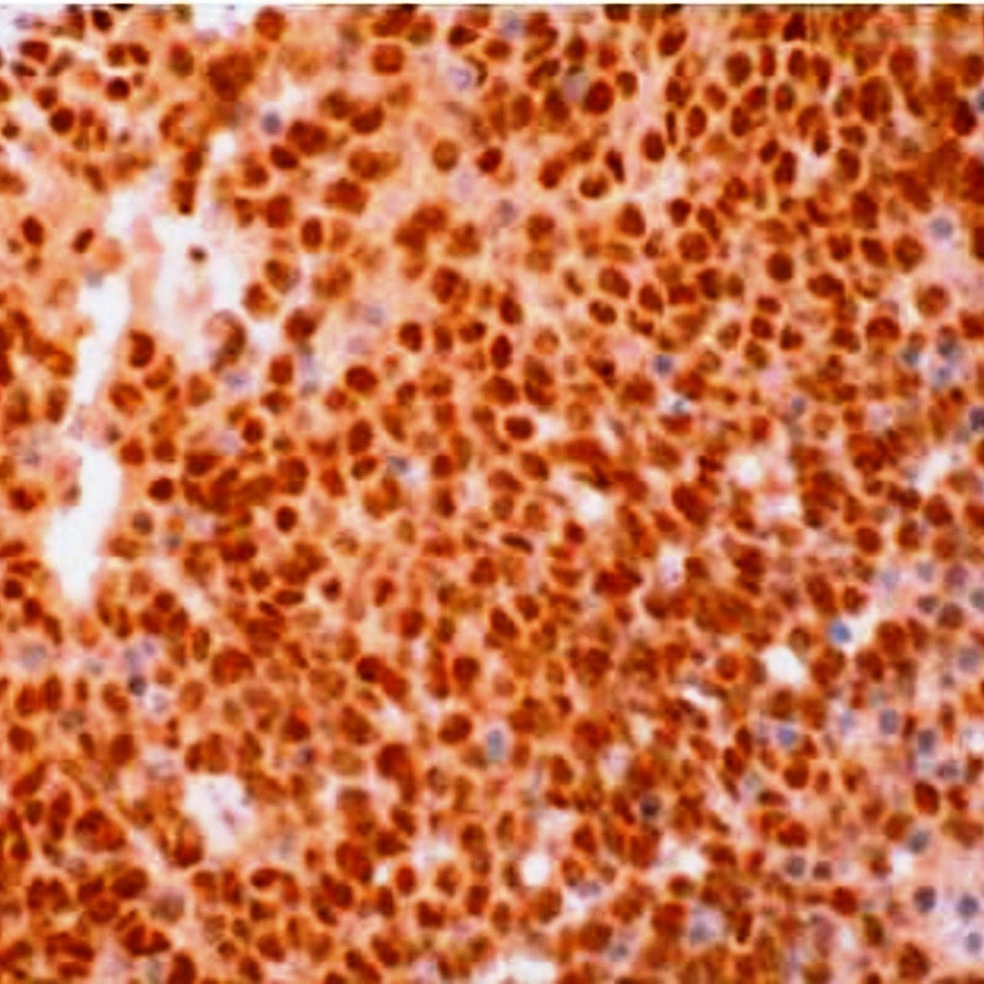
Immunohistochemistry Immunohistochemical staining of FLI-1 showing strong nuclear positivity (H&E x 200X)

**Figure 7:**
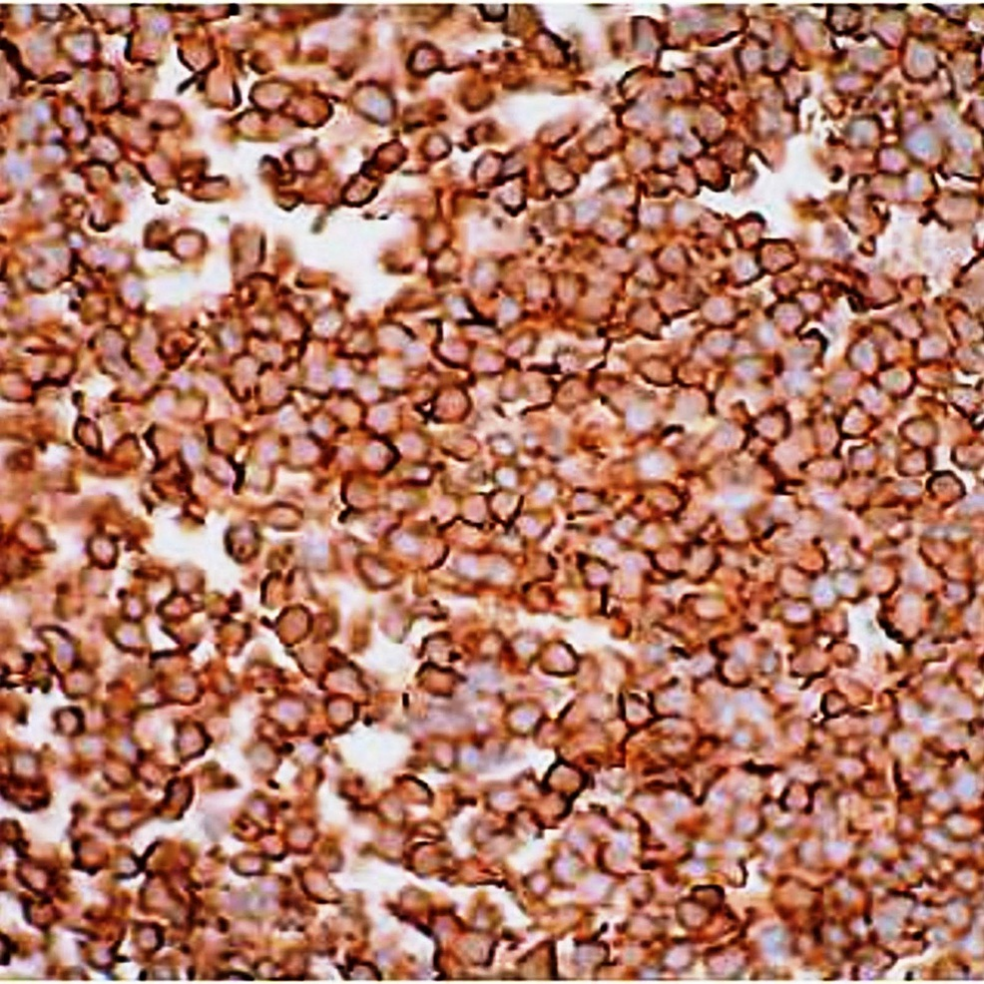
Immunohistochemistry Immunohistochemical (IHC) staining of CD99 showing strong membraneous positivity (IHC x 200X)

**Figure 8:**
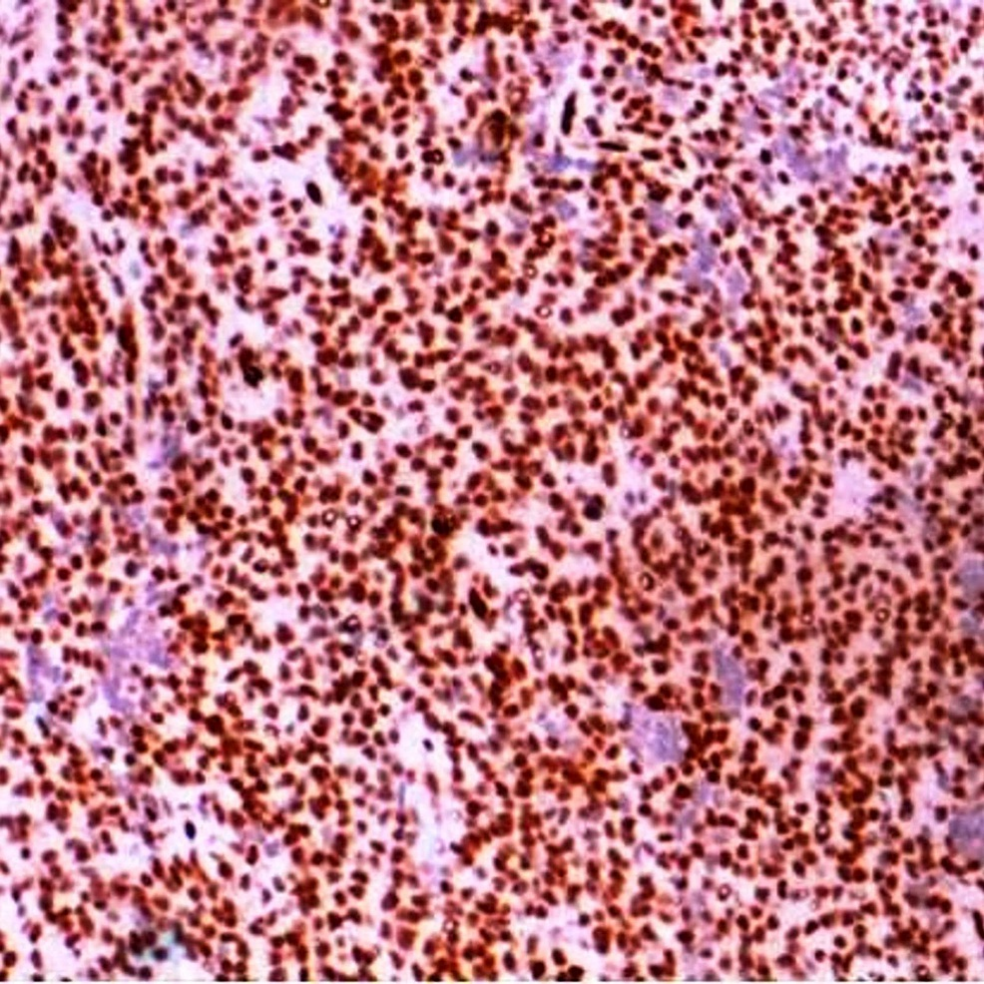
Immunohistochemistry Immunohistochemical (IHC) staining of NKX 2.2 showing strong nuclear positivity (IHC x 200X)

Out of the 58 cases of ES included in the study, 30 cases (52%) were children and adolescents (<20 years) and the rest of the 28 cases (48%) were adults (Figure 1). The mean age was 22.5 years. Female preponderance was noted, with 32 cases (56%) being females and 26 cases (44%) being males (Figure 2). The location of the tumor was variable. Twenty-five (25) cases (44%) were found in bones (Figure 3) such as the clavicle, tibia, and mandible. Seven cases were seen on the anterior chest wall. Other sites included the oropharynx, lungs, endobronchial region, infrascapular region, retroperitoneum, and thighs. One case presented as metastatic Ewings sarcoma with divergent differentiation in the lungs with the primary site of the tumor being the right humerus (Figures 4-5). Immunohistochemical studies were done on 55 of the 58 tumors. Forty-six (46) cases (83.6%) were CD99 positive (Figure 6) and 41 cases (74.5%) were FLI-1 positive (Figure 7). Eleven cases were both CD99 and FLI-1 positive. NKX2.2, a highly specific and sensitive IHC marker for ES, was positive in six cases (Figure 8).

Four of the cases were sent for NGS testing and translocation t(11:22) (EWSR-1: FLI-1) was confirmed. The demographic profile of all cases of ES is tabulated in Table 1. On follow-up of these cases, 32 cases had completed the chemotherapy protocol, had complete remission, and were on regular follow-up, eight patients were under therapy, four cases had a disease recurrence, two cases presented with distant metastasis, six cases expired, and the rest of the cases were lost to follow-up.

**Table 1:** Demographic profile

Parameters	Subcategories	Numbers (percentage %)
Age (n=58)	<20 yrs	30 (52%)
	20-40 yrs	22 (38%)
	>40 yrs	6 (10%)
Sex (n=58)	Male	26 (44%)
	Female	32 (56%)
Laterality (n=58)	Right	34 (58%)
	Left	24 (42%)
Location (n=58)	Bones	25 (44%)
	Anterior chest wall	7 (12%)
	Soft tissue	12 (21%)
	Visceral organs	5 (0.08%)
	Others	9 (16%)
Diagnostic testing (n=58)	Ultrasound or CT-guided biopsy	58 (100%)
	Excision	44 (75%)
	Others	5 (0.08%)
Size (n=58)	>8 cm	24 (42%)
	<8 cm	34 (58%)
Grade (n=58)	Low grade	24 (42%)
	High grade	34 (58%)
Immunohistochemical studies (n=55)	CD99	41 (70%)
	FLI-1	11 (19%)
	NKX2.2	6 (10%)

## Discussion

ES is the second most common bone tumor after osteosarcoma, with an annual incidence of one to three persons per million and a peak incidence in the second decade of life [7-8]. Incidence is much higher among Caucasians than African Americans and Asians [9-10]. The mean age of presentation in the present study was 22.5 years. Our study showed a female preponderance while few studies showed a male preponderance. Around 15% of ES arises in the soft tissue, whereas 25% of bony ES occurs in the pelvis and 20% occurs in the femur. If arising in the long bones, the tumor is typically located in the diaphysis [11]. The lungs are the most common metastatic site (50%), followed by bone (25%) [12]. The location of the tumor was variable in our study. Twenty-five (25) cases (43%) were found in bones such as the clavicle, tibia, and mandible. Seven cases were seen on the anterior chest wall. Other sites included the oropharynx, lungs, endobronchial, infrascapular region, retroperitoneum, and thighs. One case presented as metastatic ES with divergent differentiation in lungs with the primary site of the tumor being the right humerus.

ES presents clinically as a localized painful enlarging mass sometimes associated with pathological fracture. Systemic symptoms like fever, weight loss, anemia, leukocytosis, and increased sedimentation rate may be seen in some patients. ES, in radiology, appears as an osteolytic permeative lesion with poorly defined margins and a moth-eaten appearance indicating bone destruction. An aggressive periosteal reaction appears as the classic “onion skin appearance.” Rare cases may have a periosteal sunburst reaction but are not as common as osteosarcoma. PET is a highly sensitive and specific valuable tool for staging and restaging ES.

In resected specimens, ES grossly appears as a gray tan mass with infiltrating borders, more commonly as an intramedullary mass with soft tissue involvement. Areas of necrosis and hemorrhage are frequent. The extent of chemotherapy-induced tumor necrosis is indicative of prognosis. Microscopically, classical ES is composed of sheets of uniform small round blue cells with moderate to scant cytoplasm and round nucleus exhibiting finely stippled chromatin and inconspicuous nucleoli. The sheet-like growth pattern is separated by fibrous tissue. Few subsets show neuroectodermal differentiation exhibiting Homer wright pseudorosettes. The glycogen in the cytoplasm can be highlighted by periodic acid Schiff stain. Atypical ES shows an enlarged nucleus with irregular nuclear contours, coarse chromatin, and prominent nucleoli. Adamantinoma-like ES has nests of basaloid cells with peripheral palisading and myxoid stroma.

Immunohistochemically, 90-95% of ES shows a strong diffuse membraneous expression of CD-99. FLI-1 nuclear staining is noted in 90% of cases. Nuclear staining of ERG is seen in few cases. NKX2.2 is highly specific for ES. Adamantinoma-like ES shows a diffuse positivity for cytokeratin, p63, or p40. The most common translocation is t(11;22)(q24;q12), resulting in EWSR1-FLI1 fusion. The second most common is t(21;22)(q22;q12), resulting in EWSR1-ERG fusion. Molecular confirmation aids in reducing the error rate of diagnosing and differentiating ES from other small round blue cell tumors.

Differentiating other small round blue cell tumors from ES is significant for a definitive diagnosis. Mesenchymal chondrosarcoma is the most common differential since both have small round blue cells. Areas of hyaline cartilage differentiation and hemangiopericytoma-like vasculature are microscopic differentiating features. Also, mesenchymal chondrosarcoma exhibits HEY1-NCOA2 gene fusion. Alveolar rhabdomyosarcomas are positive for Desmin, Myogenin, and Myo-D1. They are associated with PAX3-FOXO1 and PAX7-FOXO1 gene fusion. Small cell osteosarcomas have malignant osteoid and SATB2 positivity. Primary bone lymphomas are positive for CD-45. Poorly differentiated synovial sarcomas have variable and diffuse cytokeratin positivity and strong nuclear TLE-1 expression. SSX-SS18 is the associated gene fusion. Desmoplastic small round cell tumors are positive for WT-1 and are negative for CD99. High-grade neuroendocrine carcinomas are positive for synaptophysin and chromogranin and are negative for CD-99. Round cell sarcomas with EWSR-1 non-ETS fusion have other gene fusions like EWSR1-NFATC2, FUS-NFATC2, and EWSR1-PATZ1.

NGS has become an important diagnostic adjunct to traditional morphology and immunohistochemistry for small round cell tumors [13]. Unknown ES fusion transcripts not detected by routine fluorescence in situ hybridization (FISH)/reverse transcription-polymerase chain reaction (RT-PCR) are revealed by NGS techniques. They have also discovered distinct Ewing-like tumors with different fusion partners, like Bcl6 corepressor (BCOR), and capicua transcriptional repressor (CIC)-rearranged sarcomas. BCOR-rearranged sarcomas have a round-cell or spindle-cell morphology with a prominent myxoid stroma. They harbor fusion transcripts from BCOR gene fusions with MAML3, CCNB3, or ZC3H7B genes [14-15]. BCOR-rearranged sarcomas arise predominantly in older children and young adults. They exhibit a male predominance and have a treatment protocol and prognosis similar to ES [16-17]. DUX4 and DUX4L are the most common gene fusion partners associated with CIC-rearranged sarcomas. These sarcomas display a round-cell cytomorphology with myxoid stroma. The third and fourth decades are commonly affected, and they arise predominantly in soft tissues. ETV-4 and WT-1 are positive in CIC-rearranged sarcomas, and they are negative for NKX2.2. Prognosis is less favorable as compared to BCOR-rearranged sarcomas and Ewing sarcomas [18]. CIC-rearranged sarcomas constitute 60% of round-cell sarcomas lacking EWSR1 rearrangement while BCOR sarcomas constitute only 4% [19].

The current modality of diagnosis and treatment includes a multidisciplinary team involving a radiologist, pathologist, medical oncologist, surgical oncologist, and radiation oncologist. Combinations of neoadjuvant chemotherapy are administered to reduce the tumor volume and make it feasible for surgery and reconstruction. A combination of concurrent chemotherapy and radiotherapy are effective treatment options for patients with unresectable tumors. Multiagent chemotherapy regimens, including cyclophosphamide, ifosfamide, etoposide, doxorubicin, actinomycin, and vincristine, have been proved to be effective in patients with ES. Tumors with R1 surgery can be administered radiation therapy, as ES is extremely radiosensitive [20]. The presence of >90% chemotherapy-induced tumor necrosis in resected specimens indicates a good prognosis. Metastatic, recurrent, and chemotherapy-resistant diseases, have poor outcomes. Drugs targeting the EWS-FLI1 gene, including several multikinase inhibitors, poly(ADP-ribose) polymerase (PARP) inhibitors, and mithramycin are under clinical trials [21]. ES has the potential to recur after several years of initial remission, hence prolonged surveillance is needed. Also, survivors of ES are prone to develop therapy-related late effects, so long-term follow-up care is recommended for early diagnosis.

## Conclusions

The present study on the demographic profile of ES showed a female preponderance in contrast to other studies and the mean age of presentation was slightly higher. Differentiating ES from other small round blue cell tumors needs the aid of molecular technique, as there is a diagnostic dilemma histologically and immunohistochemically. Understanding the current updates in the pathobiology, diagnosis, and treatment protocols of ES not only aids in improving the management of ES but also emphasize that clinicians should recognize and differentiate this entity from other soft tissue sarcomas with rapid progression since early diagnosis and timely treatment of ES are pivotal for a favorable oncological outcome.

## References

[1] Jaffe R, Santamaria M, Yunis EJ, Tannery NH, Agostini RM Jr, Medina J, Goodman M (1984). The neuroectodermal tumor of bone. Am J Surg Pathol.

[2] Angervall L, Enzinger FM (1975). Extraskeletal neoplasm resembling Ewing's sarcoma. Cancer.

[3] Stiller CA, Bielack SS, Jundt G, Steliarova-Foucher E (2006). Bone tumours in European children and adolescents, 1978-1997. Report from the Automated Childhood Cancer Information System project. Eur J Cancer.

[4] Zucman J, Delattre O, Desmaze C (1992). Cloning and characterization of the Ewing's sarcoma and peripheral neuroepithelioma t(11;22) translocation breakpoints. Genes Chromosomes Cancer.

[5] Ambros IM, Ambros PF, Strehl S, Kovar H, Gadner H, Salzer-Kuntschik M (1991). MIC2 is a specific marker for Ewing's sarcoma and peripheral primitive neuroectodermal tumors. Evidence for a common histogenesis of Ewing's sarcoma and peripheral primitive neuroectodermal tumors from MIC2 expression and specific chromosome aberration. Cancer.

[6] Fellinger EJ, Garin-Chesa P, Triche TJ, Huvos AG, Rettig WJ (1991). Immunohistochemical analysis of Ewing's sarcoma cell surface antigen p30/32MIC2. Am J Pathol.

[7] Esiashvili N, Goodman M, Marcus RB Jr (2008). Changes in incidence and survival of Ewing sarcoma patients over the past 3 decades: Surveillance Epidemiology and End Results data. J Pediatr Hematol Oncol.

[8] Ginsberg JP, Goodman P, Leisenring W (2010). Long-term survivors of childhood Ewing sarcoma: report from the childhood cancer survivor study. J Natl Cancer Inst.

[9] Jawad MU, Cheung MC, Min ES, Schneiderbauer MM, Koniaris LG, Scully SP (2009). Ewing sarcoma demonstrates racial disparities in incidence-related and sex-related differences in outcome. An analysis of 1631 cases from the SEER database, 1973-2005. Cancer.

[10] Worch J, Cyrus J, Goldsby R, Matthay KK, Neuhaus J, DuBois SG (2011). Racial differences in the incidence of mesenchymal tumors associated with EWSR1 translocation. Cancer Epidemiol Biomarkers Prev.

[11] Grier HE, Krailo MD, Tarbell NJ (2003). Addition of ifosfamide and etoposide to standard chemotherapy for Ewing's sarcoma and primitive neuroectodermal tumor of bone. N Engl J Med.

[12] Grier HE (1997). The Ewing family of tumors. Ewing’s sarcoma and primitive neuroectodermal tumors. Pediatric Clin N Am.

[13] Carter CS, Patel RM (2019). Important recently characterized non-Ewing small round cell tumors. Surg Pathol Clin.

[14] Kao YC, Owosho AA, Sung YS (2018). BCOR-CCNB3 fusion positive sarcomas. A clinicopathologic and molecular analysis of 36 cases with comparison to morphologic spectrum and clinical behavior of other round cell sarcomas. Am J Surg Pathol.

[15] Pierron G, Tirode F, Lucchesi C (2012). A new subtype of bone sarcoma defined by BCOR-CCNB3 gene fusion. Nat Genet.

[16] Cohen-Gogo S, Cellier C, Coindre JM (2014). Ewing-like sarcomas with BCOR-CCNB3 fusion transcript: a clinical, radiological and pathological retrospective study from the Société Française des Cancers de L'Enfant. Pediatr Blood Cancer.

[17] Puls F, Niblett A, Marland G (2014). BCOR-CCNB3 (Ewing-like) sarcoma. A clinicopathologic analysis of 10 cases, in comparison with conventional Ewing sarcoma. Am J Surg Pathol.

[18] Antonescu CR, Owosho AA, Zhang L (2017). Sarcomas with cic-rearrangements are a distinct pathologic entity with aggressive outcome. A clinicopathologic and molecular study of 115 cases. Am J Surg Pathol.

[19] Sankar S, Lessnick SL (2011). Promiscuous partnerships in Ewing's sarcoma. Cancer Genet.

[20] Ozaki T (2015). Diagnosis and treatment of Ewing sarcoma of the bone: a review article. J Orthop Sci.

[21] Arnaldez FI, Helman LJ (2014). New strategies in Ewing sarcoma: lost in translation?. Clin Cancer Res.

